# Glycosylation States on Intact Proteins Determined by NMR Spectroscopy

**DOI:** 10.3390/molecules26144308

**Published:** 2021-07-16

**Authors:** Audra A. Hargett, Aaron M. Marcella, Huifeng Yu, Chao Li, Jared Orwenyo, Marcos D. Battistel, Lai-Xi Wang, Darón I. Freedberg

**Affiliations:** 1 Center for Biologics Evaluation and Review, Laboratory of Bacterial Polysaccharides, Food and Drug Administration (FDA), Silver Spring, MD 20993, USA; Audra.Hargett@fda.hhs.gov (A.A.H.); ammarcella.7@gmail.com (A.M.M.); huifeng.yu@fda.hhs.gov (H.Y.); marcos.battistel@fda.hhs.gov (M.D.B.); 2 Department of Chemistry and Biochemistry, University of Maryland, College Park, MD 20742, USA; chaoli@umd.edu (C.L.); nyabutoo@gmail.com (J.O.); wang518@umd.edu (L.-X.W.)

**Keywords:** glycosylated proteins, heteronuclear NMR, HSQC-TOCSY, natural abundance, T_2_ filter, glycoprotein

## Abstract

Protein glycosylation is important in many organisms for proper protein folding, signaling, cell adhesion, protein-protein interactions, and immune responses. Thus, effectively determining the extent of glycosylation in glycoprotein therapeutics is crucial. Up to now, characterizing protein glycosylation has been carried out mostly by liquid chromatography mass spectrometry (LC-MS), which requires careful sample processing, e.g., glycan removal or protein digestion and glycopeptide enrichment. Herein, we introduce an NMR-based method to better characterize intact glycoproteins in natural abundance. This non-destructive method relies on exploiting differences in nuclear relaxation to suppress the NMR signals of the protein while maintaining glycan signals. Using RNase B Man5 and RNase B Man9, we establish reference spectra that can be used to determine the different glycoforms present in heterogeneously glycosylated commercial RNase B.

## 1. Introduction

Glycosylation is one of the most common post-translational modifications (PTM). There are two main types of glycosylation: (i) *O*-linked glycosylation, in which glycans are covalently linked to the hydroxyl oxygen of serine (S) or threonine (T) residues [1,2], and (ii) *N*-linked glycosylation, where glycans are attached to asparagine (N) residues within the N-X-S/T sequon [3,4,5]. In *N*-linked glycosylation, the initial glycan moiety, Glc_3_Man_9_GlcNAc_2_, is transferred to the nascent polypeptide chain co-translationally in the ER, and then the initial glycan is processed in the ER and Golgi apparatus resulting in either a high-mannose, hybrid, or complex type N-glycan (Figure S1). Because protein glycosylation is not template driven, it is inherently heterogeneous, with several factors contributing to the final glycan structure, such as protein structure [6,7], enzyme protein levels [8], Golgi transport mechanism [9], and secretory protein load [10]. Overall, this process yields heterogeneously glycosylated proteins, such as IgG, which has 32 possible glycans for its one *N*-linked glycosylation site at N297 [11].

For many glycoproteins, the glycans are critical to the protein’s structure, stability, and function [12,13,14]. For example, monoclonal antibodies (mAbs) that lack core fucose in the Fc region (remote to the antigen binding site) lead to an increase in antibody-dependent cell-mediated cytotoxicity [15,16,17,18]. IgG sialylation has been linked to anti-inflammatory activity [19]. The loss of some HIV-1 gp120 glycans leads to an increase in protein degradation and a decrease in binding to the host cell receptor [20,21,22]. In Hepatitis C virus envelope 2 protein, the loss of either N2 or N4 glycan results in total loss of HCV infectivity [23]. These are just a few glycoproteins where the location and type of glycan are critical to protein function. Thus, developing tools to characterize intact glycoproteins will aid in the understanding of optimal glycosylation for a given function, especially in protein therapeutics.

To improve our understanding of structure/function and to ensure proper glycosylation of protein therapeutics, the glycans must be fully characterized. Typically, mass spectrometry (MS)-based methods are combined with other methods, such as glycan enrichment, affinity separation, enzymatic digestion, liquid chromatography (LC) and/or gas chromatography (GC), to determine protein glycosylation [24,25,26]. However, the stereochemistry of a glycan, including the type of glycosidic linkage, are challenging to determine by MS, because it is difficult to distinguish between isobaric species like glucose (Glc), galactose (Gal), and mannose (Man). To overcome these limitations, a direct, robust and simple NMR spectroscopy method was recently proposed for the detection and identification of protein glycoforms by denaturing the glycoprotein in urea [24]. This method provides a significant advantage by indirectly detecting modifications on intact proteins without sophisticated sample preparation or isotopic labeling. Moreover, the method is not limited by the protein’s molecular weight due to the more favorable nuclear relaxation properties of denatured proteins. RNAse A and RNase B have identical amino acid sequences, but RNase B is glycosylated. In this report, we show that the glycans in intact folded RNase B can be characterized by NMR spectroscopy.

As a proof of concept, we chose RNase B glycoprotein as a model system because it is characterized by the following key properties: it is a ~15 kDa glycoprotein, a size that is amenable to NMR and enables the study of native glycosylation; it contains a single glycosylation site at N34 yet, it exists as five glycosylated variants (Man_5-9_GlcNAc_2_) and therefore, RNase B permits the study of the potential microheterogeneity in a single glycosylation (at N34) [27]; finally, previous studies of RNase B can be used to cross-validate our findings. In a study of commercial RNase B, the oligosaccharides were released and isolated, and the relative molar portions of Man_5_ to Man_9_ were determined to be 57, 31, 4, 7, and 1%, respectively [28].

In pioneering work, Brown showed that differential T_2_s can be used to distinguish between fluids with different viscosities [29]. Herein, we build on this idea, using ^1^H-^13^C HSQC-TOCSY [30,31], with varied mixing times on natural abundance samples for fast detection and analysis of glycoprotein microheterogeneity, without complicated sample preparation. The mixing time efficiently relaxes away protein resonances and, although this phenomenon is not unexpected, it hasn’t been investigated in detail [32]. In this report, we show that using a T_2_ filter in small glycoproteins reduces the spectral complexity that arises from the protein peaks yet captures the glycosylation microheterogeneity by retaining glycan peaks.

## 2. Results and Discussion

### 2.1. ^1^H-^15^N HSQC of RNase A and RNase B

^1^H-^15^N HSQC spectra of unlabeled RNase A and RNase B were collected at 700 MHz, 37 °C in 5 h with all expected signals, consistent with previous results [33]. Peaks were assigned based on ^1^H-^15^N chemical shifts deposited in the Biological Magnetic Resonance Data Bank (BMRB) for RNase A. Like mapping protein ligand binding sites by comparing apo and bound forms’ ^1^H-^15^N chemical shift changes, protein backbone amino acid chemical shifts can be affected by PTMs. Backbone resonance assignments of RNase A/B provide useful data that were used to identify the effect of glycosylation on the polypeptide chain. Upon glycosylation, the backbone ^1^H-^15^N chemical shift perturbation in RNase B compared to RNase A is confined to the region around the glycosylation site (±4 amino acids, Figure S2). Minimal changes were observed for most of the glycoprotein’s NMR signals. However, measurable differences were observed at N34 (glycosylation site). Specifically, T36 shifts 0.064 ppm in ^1^H, and S32, N34, and K37 change by 0.014 ppm in ^1^H. In ^15^N, S32, N34, T36, and K37 change by 0.28, 0.93, 0.63, and 0.28 ppm, respectively. Interestingly, R33 is absent in the RNase B spectrum, and L35′s chemical shift is unchanged. Thus, in RNase B only polar or charged residues proximal to the glycosylation site exhibit a change in chemical shift. It may also be that both charged residues and N34, the glycosylated residue, are exposed, thus when N34 is glycosylated, other exposed residues are affected. While these ^1^H-^15^N spectra suggest that PTMs effect the protein and the location of attachment, they do not provide accurate information regarding the precise identity of the modification.

### 2.2. ^1^H-^13^C HSQC of RNase B Man5 and RNase B Man9

In contrast to natural abundance ^1^H-^15^N HSQC, natural abundance ^1^H-^13^C HSQC spectra are higher in sensitivity and can provide substantially more information regarding protein glycosylation. The larger number of ^13^C atoms in an amino acid than ^15^N atoms increases spectral complexity; nevertheless, the uniqueness of ^13^C chemical shift ranges and NMR experiments can be used to differentiate between protein and glycan subspectra.

**Figure 1:**
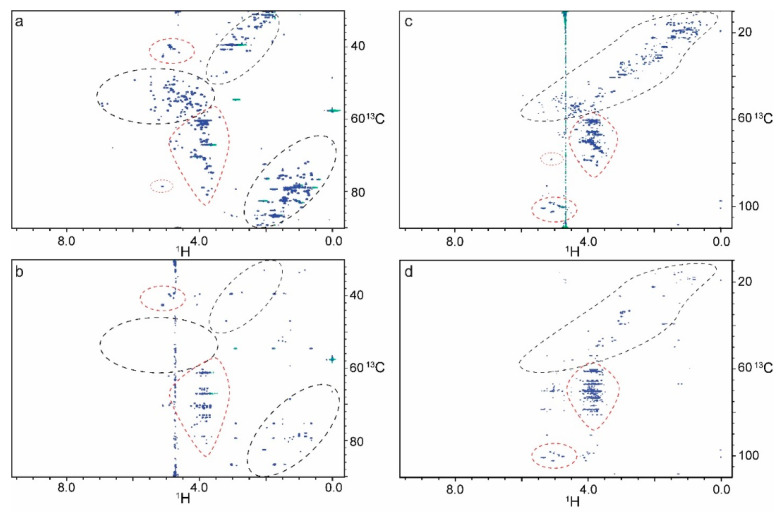
Comparison of ^1^H-^13^C HSQC and HSQC-TOCSY spectra of RNase B Man_5_ and Man_9_. (**a**) HSQC spectrum of 0.3 mM RNase B Man_5_ contains peaks for both protein (black circles) and glycans (red circles). RNase B Man_5_ anomeric protons (upper red circle) are folded in the ^13^C dimension and range from 98–103 ppm. (**b**) An HSQC-TOCSY with a 90 ms mixing time of RNase B Man_5_ takes advantage of the longer glycan T_2_, so that the glycans peaks are retained while the protein peaks are greatly reduced. Similarly, an HSQC of 0.6 mM RNase B Man_9_ (**c**) shows peaks for both protein and glycan, while in the HSQC-TOCSY experiment (**d**) mostly glycan peaks are retained.

**Figure 2:**
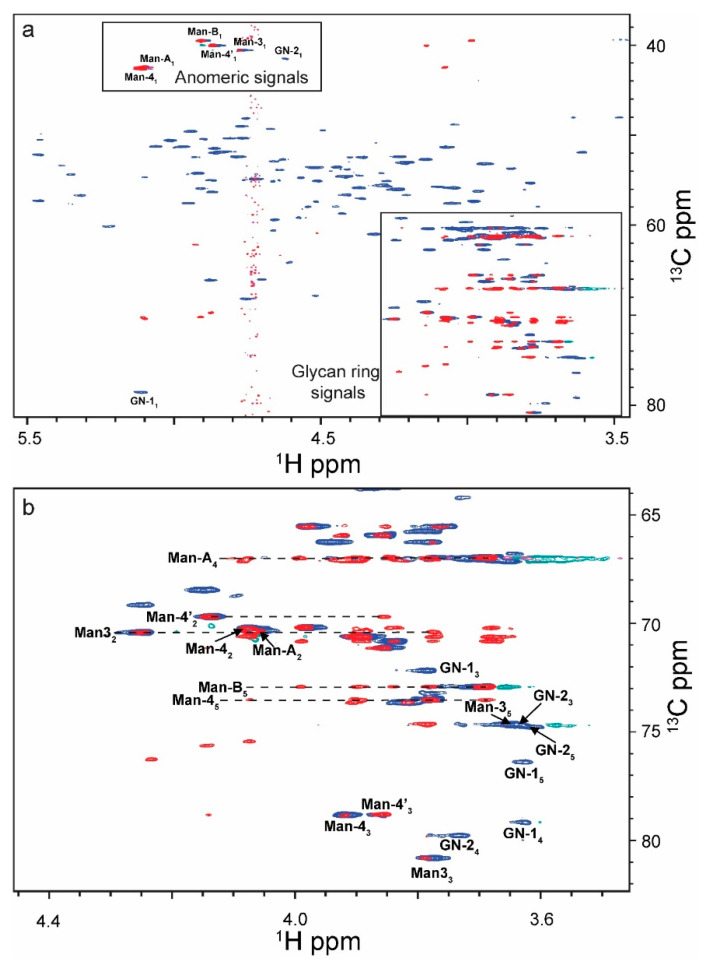
Overlay of a 2D ^1^H-^13^C HSQC (blue) and ^1^H-^13^C HSQC-TOCSY (red) of 0.3 mM RNase B Man_5_ at pH 6, 37 ^o^C (blue) and ^1^H-^13^C HSQC-TOCSY (red) (**a**). Chemical shifts of ^13^C anomeric signals are folded and range from 98 to 104 ppm, except GlcNAc_1_ which is more shielded with a ^13^C chemical shift of 78 ppm. (**b**) Glycan ring signals with lines drawn to show the 90 ms TOCSY correlations for each of the monosaccharides except GlcNac_1_ (GN1) and GlcNAc_2_ (GN2).

**Figure 3:**
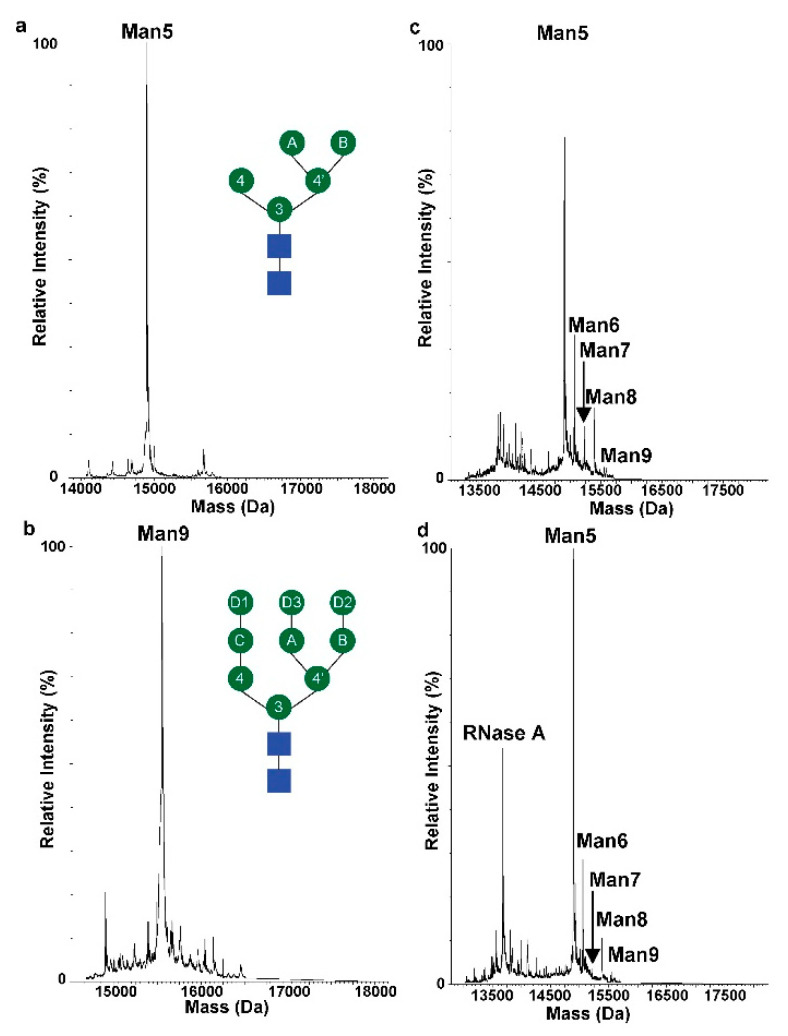
Deconvoluted ESI-MS spectra of (**a**) RNase B Man_5_, (**b**) RNase B Man_9_, (**c**) vendor 1 RNase B, and (**d**) vendor 2 RNase B. RNase B Man_5_ and RNase B Man_9_ are singly glycosylated. Both vendor 1 and vendor 2 contain a population of high-mannose glycan with predominantly GlcNAc2Man5. The vendor 2 sample did have a significant population of RNase A that was not present in Vendor 1’s sample.

Glycan anomeric ^1^H-^13^C correlations occur in a unique spectral region which does not overlap with most protein signals [24,34], since both nuclei are typically deshielded in ^1^H (4.3–5.8 ppm) and ^13^C (98–106 ppm). In the case of pure glycans or single glycoforms, the number of anomeric peaks can be used to determine the number of saccharide residues in a given glycan. ^1^H-^13^C HSQC spectra were taken of two engineered RNase B glycoproteins (Figure 1 and Figure 2), each uniformly glycosylated at N34 with either Man_5_GlcNAc_2_ (Man_5_) or Man_9_GlcNAc_2_ (Man_9_). Intact electrospray ionization mass spectrometry (ESI-MS) showed that each glycoprotein contained one predominant mass after charge state deconvolution (14898 Da, RNase B Man_5_; 15546 Da, RNase B Man_9_, Figure 3). Figure S1 provides schematics of different types of glycan and their linkages. For RNase B Man_5_, seven anomeric peaks were unambiguously observed (Figure S3a). GlcNAc2, Man3, Man4, Man4′, ManA, and ManB have ^13^C chemical shifts between 100 and 144 ppm and ^1^H chemical shifts between 4.6 and 5.2 ppm. The GlcNAc_1_ anomeric peak is shifted significantly, in ^13^C, to 78.4 ppm as it is amide linked to the protein [24]. Notably, there are no overlapping protein chemical shifts in this region.

In a ^1^H-^13^C HSQC of RNase B Man_9_, anomeric correlations are observed in a similar spectral region as RNase B Man_5_ (Figure S3b). However, because RNase B Man_9_ contains additional Man residues D1-D3, with α1-2 linkages, their signals overlap and were resolved with Lorentz-to-Gauss processing for line narrowing [35]. RNase B Man_9_ provides an additional challenge because the NMR signals for the C2-C6 positions on each glycan significantly overlap. Although the spectra were collected at 34 Hz/pt ^13^C resolution, it is still insufficient to resolve the individual signals within the ring. Nevertheless, the unique fingerprint in the anomeric region is the ideal method for distinguishing glycoforms. The most evident signals are those belonging to ManA (+D3), ManC (+D1), ManB (+D2) and Man4 (+C). These three signals are unique to Man_9_, as Man_5_ does not contain a ‘C’ residue and ManA and Man4 are more deshielded in the ^1^H dimension when linked to the D mannoses. A similar strategy was used to characterize the glycoprofile of FcεRiα [36]. These researchers were able to assess the different glycoforms using HSQC spectra of uniformly ^15^N/^13^C-labeled glycoproteins under both folded and denatured sample conditions at lower concentrations than we report. They also report assessing the relative abundances of each glycoform using the anomeric region only. Thus, both their methods and those we report here can be used to assess glycoforms.

### 2.3. Relaxation Selection for Glycan Regions of Spectrum

To enrich glycan regions of the spectrum for peak assignment and reduce ambiguity observed in ring regions of the spectrum, ^1^H-^13^C HSQC-TOCSY experiments were used [30,31]. TOCSY mixing times were optimized by a simple linewidth analysis. Data collected at a sufficient resolution to obtain reliable linewidths in both ^1^H and ^13^C dimensions can be used to estimate the upper limits of the T_2_s, using the relation (T2≈(π∗(Δν12))−1, where Δν12 is the linewidth at half height [37]. Table S1 shows a list of peaks corresponding mostly to either the ring or anomeric region of the N-glycan or ^1^H, ^13^Cα peaks from the protein. Based on linewidths, the range of ^13^C transverse relaxation times for the protein specific regions is 9–13 ms with an average of 11.6 ms, whereas in the glycan regions the T_2_ range is 12 to 17 ms and an average of 14.4 ms. This yields an approximate difference in relaxation time of 25% between the glycan and protein components, limiting the amount of relaxation effect to exploit. In contrast to the ^13^C relaxation times, ^1^H relaxation times displayed a greater disparity between the protein and glycan resonances. The protein-specific relaxation times in ^1^H were between 10 and 35 ms, with an average of 16.4 ms. The glycan relaxation times in ^1^H ranged from 14 to 45 ms and averaged 29 ms. This provides a nearly twofold (80%) difference in relaxation times which is easier and more effective to exploit. The average T_2_ determined from this analysis was then used to plot transverse magnetization loss over time (Figure S4). This allows for quantitatively selecting mixing times to maximize the intensity difference between the protein and glycan peaks. Because the relaxation rate difference is nearly twofold, it allows most of the protein signals to relax while maintaining enough glycan signal so as not to increase experiment time.

Signal-to-noise ratios (SNR) in protein dominant regions (2a/2b) and glycan dominant regions (1a/1b) were assessed in an HSQC and HSQC-TOCSY of RNase B Man_5_ (Table 1, Figure S5). The glycan regions maintain 43.3% of their signal intensity in the HSQC-TOCSY (90 ms mixing time), compared to the HSQC, where in the protein regions only an average of 11.8% of the initial intensity remains. This 3.7-fold difference agrees with the estimated signal loss calculated using the relaxation times (3.2-fold) and significantly simplifies the spectra while also providing the benefit of intra-ring correlations of coupled ^1^Hs through the TOCSY (Figure 1 and Figure 2). Interestingly, signal loss is observed for glycan residues GlcNAc1 and GlcNac2 which are spatially close to the protein and have a T_2_ closer to that of the protein C_α_. Other NMR experiments such as the HSQC-ROESY have a similar effect on protein signal attenuation.

### 2.4. Analysis of Commercial RNase B Samples

Using the uniformly glycosylated RNase glycoproteins as references, two commercially available RNase B samples were evaluated. RNase B from vendor 1 was reported to be 80% pure, and RNase B from vendor 2 was reported to be 50% pure. All RNase B samples were analyzed for glycosylation heterogeneity and purity using ESI-MS. Mass spectra of intact RNase B were collected and a charge envelope consisting of +8 to +15 charged ions were observed for each of the samples. The charge state envelope was deconvoluted [38] using the Waters MassLynx MS software and the glycosylation pattern was determined for each of the RNase B samples (Figure 3). The commercial RNase B from vendor 1 contained predominantly GlcNAc_2_Man_5_ at N34 (exp = 14,898 Da, calc = 14,897 Da), with a small percentage of GlcNAc_2_Man_6-9_. Similarly, commercial RNase B from vendor 2 was mostly glycosylated with GlcNAc_2_Man_5_; however, this sample also contained RNase A (exp = 13,682 Da, calc = 13,681 Da). To have similar amounts of RNase B for the NMR analysis in both vendors, the percent of RNase A was accounted for when determining RNase B sample concentration for vendor 2. Overall, the distribution of N-glycans in RNase B is similar between the two manufacturers which should lead to nearly identical samples in the NMR experiments.

**Figure 4:**
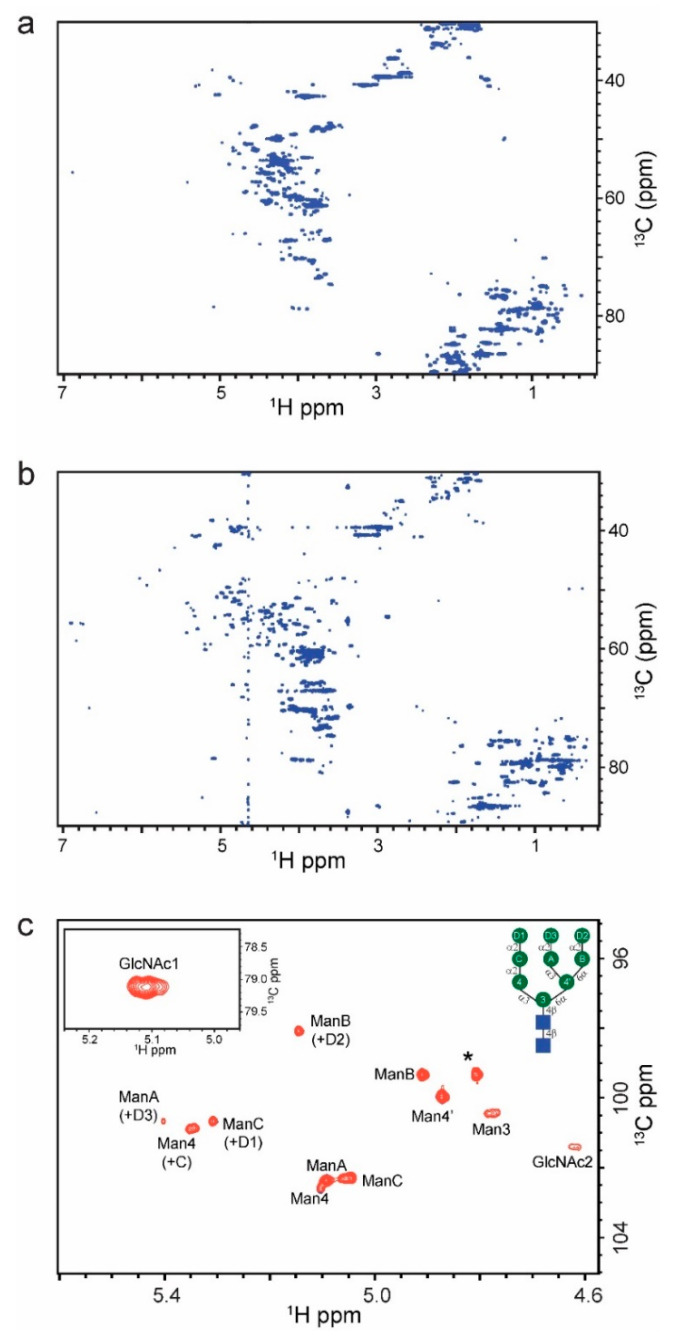
2D ^1^H-^13^C HSQC of (**a**) 1.4 mM vendor 1 RNase B and (**b**) 1.0 mM vendor 2 RNase B. Glycan anomeric region and ring region are the same between the two vendors; however, the vendor 2 spectrum contains additional peaks from 2.5 to 3.0 ppm in ^1^H and 30 to 40 ppm in ^13^C that are not present in the RNase B vendor 1 spectrum. (**c**) Vendor 1 glycan anomeric signals used for quantitative analysis (inset: schematic of Man_9_ glycan).

Initial ^1^H-^13^C HSQC analysis of the commercial RNase B revealed a contaminating peak present in vendor 1’s sample (Figure S6, blue) that was not observed in the ESI-MS analysis (data not shown). It is possible that this glycoside-like molecule is a methyl mannoside that was either not completely removed after lectin affinity chromatography or was used to stabilize RNase B. Due to similarities in chemical shift between this contaminant and the RNase B glycan and its high SNR in the HSQC-TOCSY, vendor 1 RNase B was dialyzed using a 1 kDa MWCO membrane to remove the contaminant (Figure S6, red). After the dialysis, there were only minor differences between the vendor RNase B samples (Figure 4). Specifically, RNase B from vendor 2 contained peaks from 2.5–3.0 ppm ^1^H and 30–40 ppm ^13^C that are not present in vendor 1′s RNase B. The chemical shifts that correspond to the glycan anomeric (4.5–5.5 ppm ^1^H and 95–105 ppm ^13^C) and ring regions (3.3–4.3 ppm ^1^H and 60–80 ppm ^13^C) are the same in both vendor RNase B spectra.

The ^1^H-^13^C HSQC and ^1^H-^13^C HSQC-TOCSY spectra from the vendor samples, which contained a heterogenous population of glycans (Man_5-9_GlcNAc_2_), was compared to the uniformly glycosylated reference RNase B spectra. Figure 4 shows the anomeric region of the commercially available RNase B from vendor 1 with transferred assignments from literature values [33]. In addition to the peaks observed in the RNase B Man_5_ reference spectrum, there are additional peaks corresponding to Man 4, A, B, and C as each of these positions can be further modified by an α1-2 linked mannose residue. Man D1, D2, and D3 chemical shifts overlap Man A, C, and 4 and cannot be assigned at the current spectral resolution. Thus, the ratios of the entire glycan population cannot be qualitatively estimated using these signals. Nevertheless, the SNR of some of the glycan anomeric signals can be used for quantification, as we show below.

### 2.5. Quantitative Analysis of Commercial RNase B Glycoforms

To normalize the results for quantitative analysis, all experiments performed were collected on a 700 MHz (^1^H) magnet equipped with cryoprobe, which provided increased sensitivity. This is especially useful in experiments carried out at natural abundance, as performed in the present study. One HSQC was run with nearly identical experimental conditions for the three samples analyzed (RNase B Man_5_, RNase B Man_9_, and commercial RNase B (vendor 1)), protein concentrations were between 18–22 mg/mL. The temperature was set to 25 °C for Man_5_ and commercial RNase B and 37 °C for Man_9_ RNase B. In all cases, the lowest SNR was observed for GlcNAc1 and GlcNAc2 anomeric signals. The SNRs of GlcNAc1 were standardized to account for differences in protein concentration between samples, as all experiments were performed with the same number of scans and t_1_ points. RNase B Man_9_ GlcNAc1 had a SNR of 19:1, RNase B Man_5_ GlcNAc1 had a s/n of 16:1 and commercial RNase B 16:1.

Quantitative ratios of each glycoform present in the commercial RNase B are difficult to obtain due to differential T_2_ relaxation. For example, Man C and Man 3 cannot be compared, since Man 3 is far more restricted and would be expected to have more efficient relaxation, leading to a decrease in peak intensity that would reduce accuracy in assessing relative abundance of glycoforms. Therefore, to better estimate the relative glycoforms abundance, only residues with similar T_2_s can be compared such as Man B and Man C. The ratio of Man B: Man C correlates to glycoforms GlcNAc_2_Man_5_ and GlcNAc_2_Man_6_. This ratio was determined to be 1.8:1 by peak height comparison, which is in line with the reported estimate of 1.84:1 [28]. Another ratio that should be close to 1:1 is that of ManC (+D1):ManB (+D2) as there is more GlcNAc_2_Man_8_ than GlcNAc_2_Man_7_ according to the MS analysis leading to equal amounts of the two terminal mannose residues present in GlcNAc_2_Man_8_. In this sample the ratio was 1.04:1 in line with the expectation.

## 3. Materials and Methods

RNase B from bovine pancreas (Cat. R7884) purchased from Sigma-Aldrich St. Louis, MO, USA) and VWR and all other chemicals were purchased from Sigma-Aldrich (unless otherwise noted). RNase A from bovine pancreas were from Roche (Cat. 10109142001).

### 3.1. Preparation of RNase B with Homogenous Man_5_ and Man_9_ N-Glycans

The synthesis of RNase B Man_9_ followed our previously reported method [39]. ESI-MS: theoretical mass for RNase B Man_9_, M = 15,546 Da; found (deconvolution data) (*m*/*z*) 15,547 Da. RNase B Man_5_ was prepared following a similar chemoenzymatic method. Briefly, the Man_5_ oxazoline was obtained by α1,2-mannosidease catalyzed hydrolysis of Man_9_ N-glycan, followed by Endo-A treatment to provide the Man_5_GlcNAc, which was converted to Man5GlcNAc-oxazoline by treatment with 2-chloro-1,3-dimethylimidazolium chloride (DMC) and triethylamine in water [40]. A solution of Man_5_GlcNAc-oxazoline (500 μg, 0.49 μmol) and GlcNAc-RNase (500 μg, 0.036 μmol) was incubated with EndoA-N171A (200 μg) in buffer (PBS, 100 mM, pH 7.4, 10 μL) at 30 °C for 8 h. The reaction was monitored by analytical HPLC, and the glycoprotein product was isolated by preparative HPLC to give Man_5_GlcNAc_2_-RNase as a white foam after lyophilization (418 μg, 78%). ESI-MS: calc’d. for RNase B Man_5_, M = 14,897 Da; found (deconvolution data) (*m*/*z*) 14,898 Da.

Analytical reverse-phase high-performance liquid chromatography (RP-HPLC) was performed on a Waters 626 HPLC instrument equipped with an YMC-Triart C18 column (5 μm, 4.6 × 250 mm) for reverse phase. The YMC-Triart column was eluted using a linear gradient of acetonitrile (22–29%, *v*/*v*) with water containing 0.1% TFA over 35 min at a flowrate of 0.5 mL/min under UV 280. The LC-ESI-MS was performed on an Exactive™ Plus Orbitrap mass spectrometer (Thermo Scientific) equipped with a C8 column (Poroshell 300SB-C8, 1.0 × 75 mm, 5 μm, Agilent). Mass spectra were analyzed, and deconvolution of MS data was obtained by MagTran.

### 3.2. NMR of Glycoproteins

All NMR experiments were performed at 700 MHz ^1^H Frequency (16.5 T magnet) with a Bruker Avance III HD console and 5 mm triple gradient TCI cryoprobe. RNase B solution concentrations varied from 5-20 mg/mL, depending on sample, in 20 mM phosphate buffer at pH* 6.0 with added DSS as an internal reference in ~99% D_2_O. For HSQC (Bruker pulse sequence hsqcetgpsi) and HSQC-TOCSY (Bruker pulse sequence hsqcdietgpsi) experiments, 4096 and 768 total points in ^1^H and ^13^C, respectively, were collected. Non-uniform sampling was used in the indirect dimension with 30–50% of the points collected based on the schedules from the Wagner group [41]. Data were collected with spectral windows of 7002.801 Hz (10 ppm) and 10,563.504 Hz (60 ppm), with carrier frequencies of 4.7 ppm and 60 ppm in ^1^H and ^13^C, respectively, and were reconstructed using SMILE [42]. The anomeric ^13^C peaks appear at ~40 ppm, due to folding in most of the spectra collected. Data were processed with zero-filling to 2x the total points collected in ^1^H and ^13^C. A square cosine bell window function was applied in both dimensions. For linewidth measurements, data were collected with traditional sampling and 2048 × 1024 total points in ^1^H and ^13^C, respectively. Spectral widths of 10 ppm (7002.8 Hz) in ^1^H and 100 ppm (17,605.8 Hz) in ^13^C were used with the ^13^C carrier set at 60 ppm (10,562.9 Hz). Data were processed in the same way as described above.

For ^1^H-^15^N experiments, samples of RNase B and RNase A were dissolved in 20 mM phosphate buffer pH 6.5 in 2.5% D_2_O at ~15 mg/mL (~1 mM). ^1^H-^15^N experiments were collected using an HSQC pulse sequence with a flip back pulse and WATERGATE [43] element for water suppression. Acquisition times of 41 and 26 ms for ^1^H and ^15^N respectively were used with spectral resolution of 12 and 19 Hz/pt. High-quality data were collected over ~10 h of experiment time; two 10 h spectra were added to increase signal to noise.

**ESI-MS of RNase B**: 2.5 µL of a 0.5 mg/mL solution of RNAse B was injected into a C18 trap column and eluted using a gradient from 0 to 40% acetonitrile in acetate buffer, pH 4.5, at a flow rate of 0.5 µL/min. Data were collected on a Waters Synapt G2 HDMS system with a nanoAcquity LC system. Data containing the entire charge state envelope were deconvoluted using the Masslynx software yielding the mass of the singly charged species.

**MALDI-TOF MS of RNase B**: 0.5 mg/mL RNase B was mixed 1:1 with dihydroxybenzoic acid matrix solution (10 mg/mL in 50:50 acetonitrile:water with 0.1 % TFA). The mixture was then spotted on a stainless steel MALDI target and allowed to air dry. Samples were analyzed using a Bruker Autoflex Speed instrument with a voltage of 13 keV, 4000 shots per spectrum and delay time of 800 ns. Samples were all shot in positive ion mode with singly, doubly, and triply charged states observed.

## 4. Conclusions

Multiple RNase B samples were tested using a standard set of HSQC and HSQC-TOCSY pulse sequences with varying mixing times. The size of RNase B offers a protein with favorable T_2_ relaxation times when compared to even larger proteins and even more beneficial is the expected mobility of the N-glycan. The RNase B N-glycan was not observed in the crystal structure suggesting an unrestrained conformation which allowed us to exploit the relaxation differences between the protein and glycan. This difference in protein and carbohydrate relaxation times provides the opportunity to analyze the two components of the spectra independently on increasingly complex samples. We will use these experiments to fine-tune the conditions under which NMR spectra of polysaccharide conjugate vaccines can be better analyzed.

## Figures and Tables

**Table 1:** Peak volume between glycan dominant (1a/1b) and protein dominant (2a/2b) spectral regions.

Region	^1^H (ppm)	^13^C (ppm)	HSQC Peak Volume	HSQC-TOCSY Peak Volume	% Remaining Volume
1a	4.5-5.5	37.5-45.0	1416.69	584.12	41.2
1b	3.5-4.5	60.0-80.0	14461.11	6563.13	45.4
2a	3.5-5.5	45.0-60.0	13203.96	883.33	6.7
2b	0.2-3.5	30.0-90.0	42549.51	7195.17	16.9

## Data Availability

Not applicable.

## Supplementary Materials

The following are available online, Figure S1: Schematic of N-glycans using symbol nomenclature; Figure S2: Overlay of 1H-15N HSQC of RNase A and RNase B; Figure S3: Anomeric region of the 1H-13C HSQC of uniformly glycosylated RNase B Man5 and RNase B-Man9; Figure S4: Plots of the average T2 relaxation for proteins and glycans; Figure S5: Intensity regions used in Table 1 for the 1H-13C HSQC and the HSQC-TOCSY for RNase B Man5. Table S1: Linewidths and estimated T2 relaxation values for protein Cα’s and glycan anomeric ring 1H-13C correlations.
