# Supplementary material for: Glycosylation States on Intact Proteins Determined by NMR Spectroscopy
Source: Molecules. 2021 Jul 16;26(14):4308. doi: 10.3390/molecules26144308 (PMC8303171; doi:10.3390/molecules26144308)
Supplement: Supplementary file 1 [file molecules-26-04308-s001.zip › molecules-1274642-supplementary.pdf]

Supplementary Materials

Glycosylation states on intact protein determined by NMR spectroscopy

Audra A. Hargett[1], Aaron M. Marcella[1], Huifeng Yu[1], Chao Li[2], Jared Orwenyo[2], Marcos D. Battistel[1], Lai-Xi Wang[2] and Darón I. Freedberg[1]*

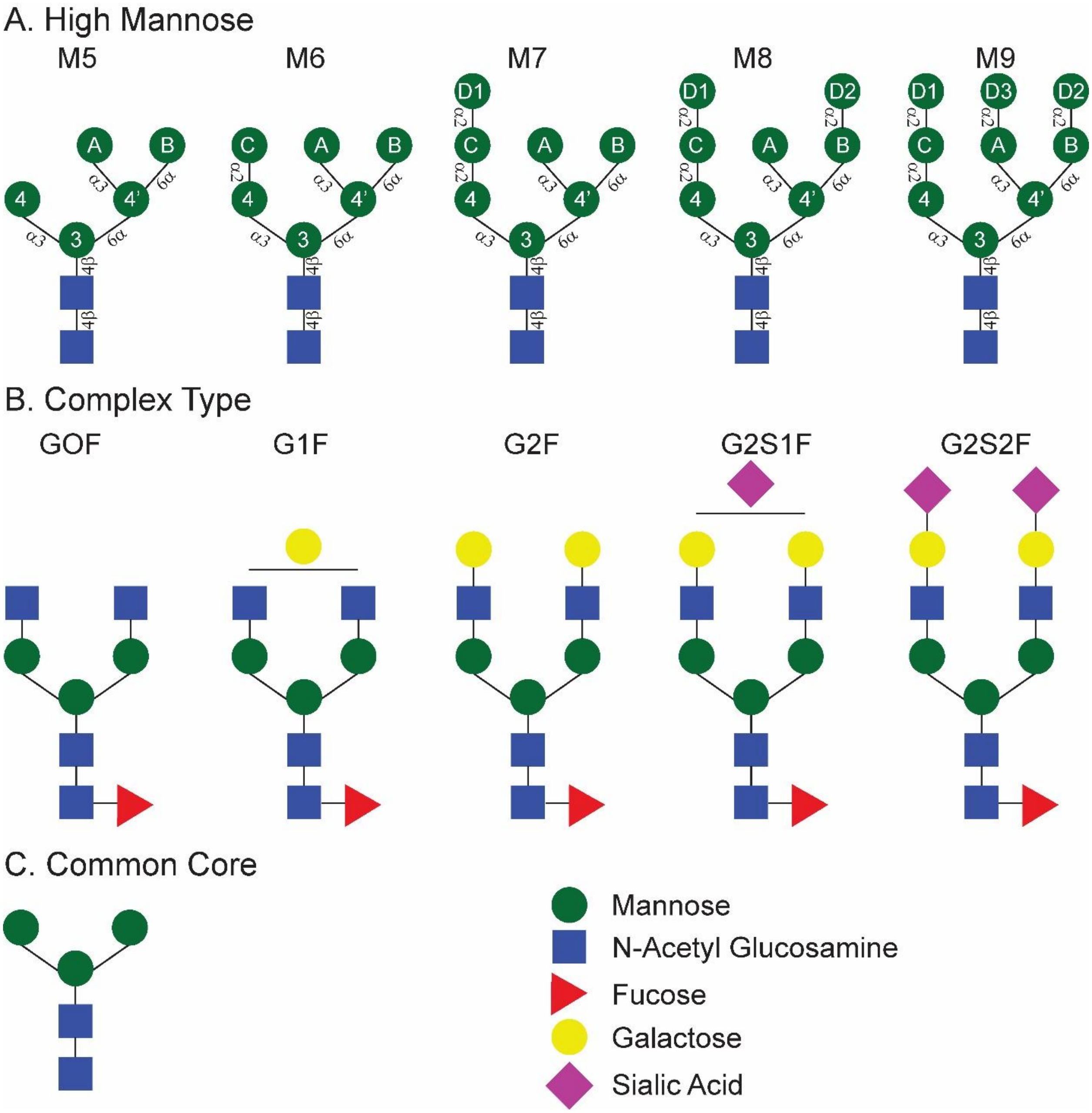

Figure S1. Schematic of *N*-glycans using symbol nomenclature and their linkages.

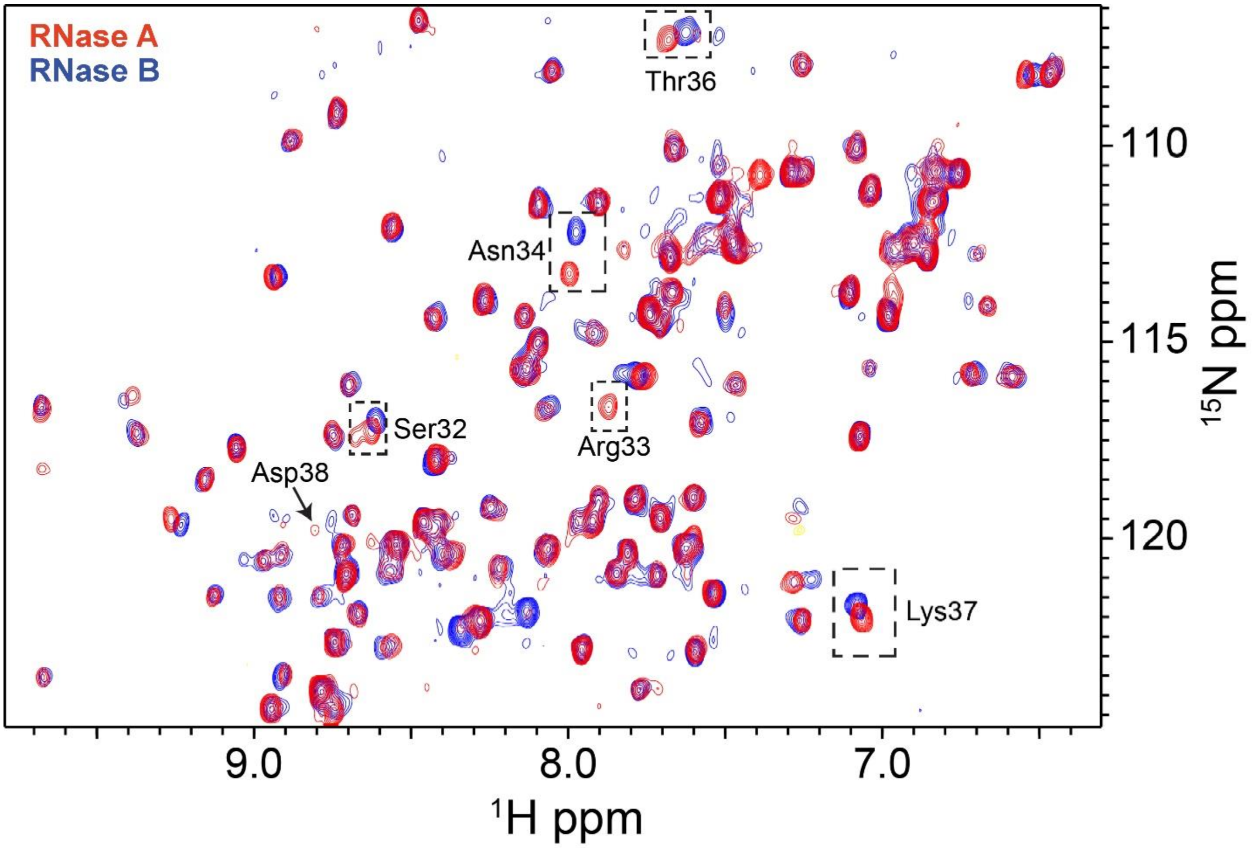

Figure S2: Overlay of ^{1}H-^{15}N HSQC of RNase A (red) and RNase B (blue) spectra. Chemical shift perturbations are observed at S32, N34, T36, and K37 when RNase A is glycosylated at N34 to become RNase B. There is a loss of the R33 signal in the RNase B spectrum. All other amino acids remain unchanged, thus only the chemical shift of polar or charged residues near the site of attachment are affected by the glycan. This data was taken at pH 6.0, 37°C at 700 MHz.

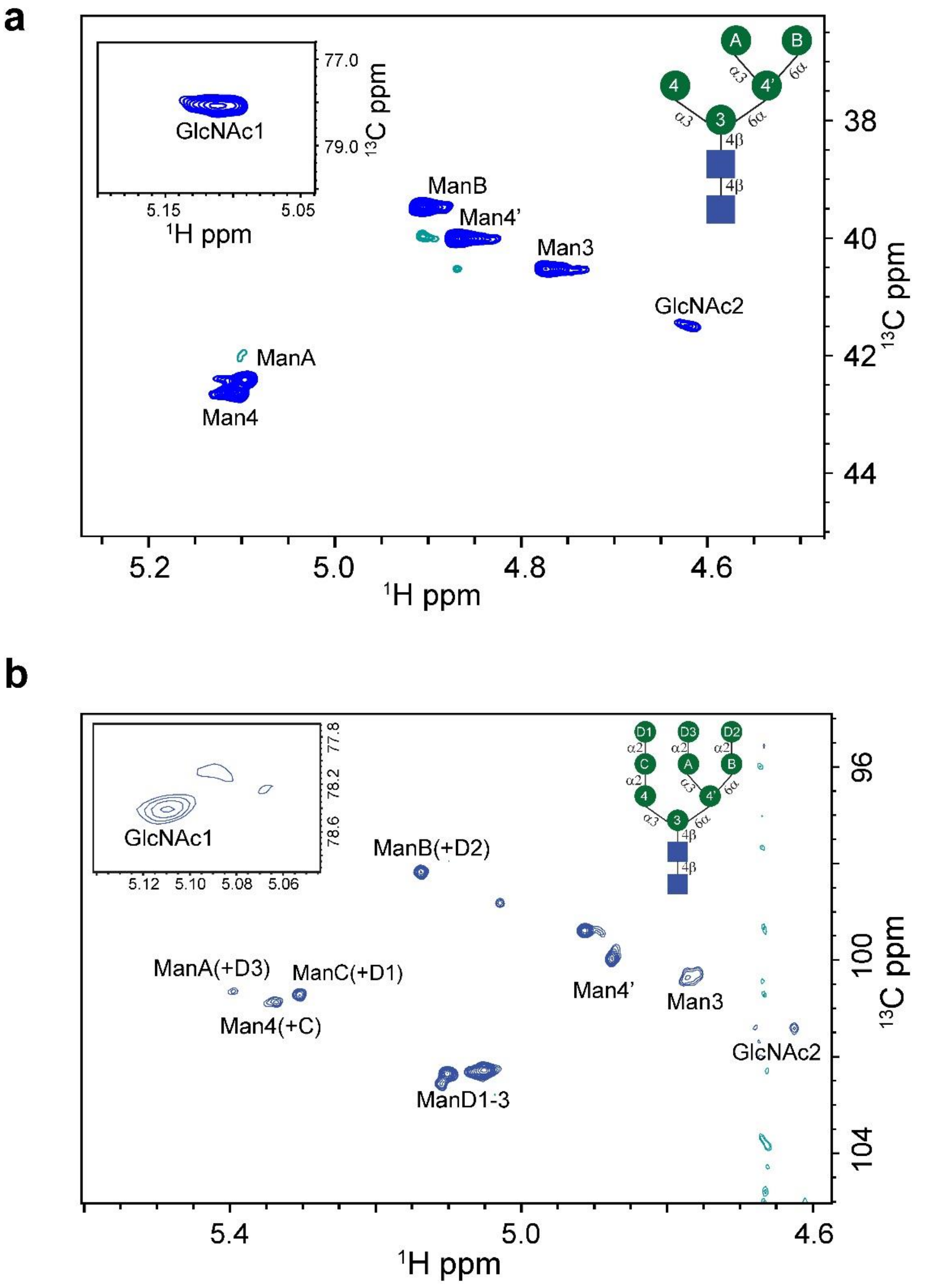

Figure S3: The anomeric region of the ^{1}H-^{13}C HSQC spectrum of uniformly glycosylated (a) RNase B Man$_5$ and (b) RNase B Man$_9$. The peaks marked with an asterisk in RNase B Man$_9$ were reduced/removed after washing the protein sample through a 10kDa Amicon™ filter.

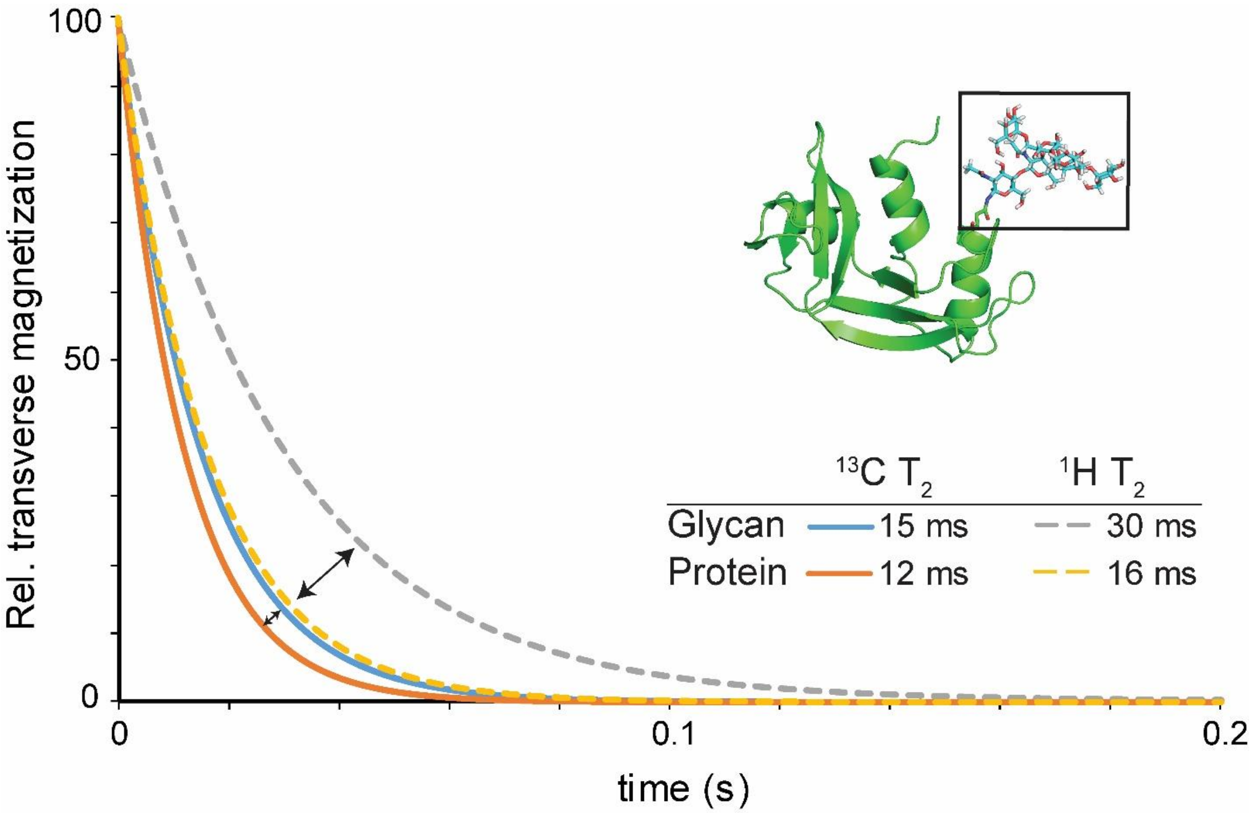

Figure S4: Plot of the average T_2 relaxation for proteins and glycans. The glycan ^{1}H (grey) and ^{13}C (blue) T_2 average relaxation times are slower than the protein C_α ^{1}H (yellow) and ^{13}C (orange) average T_2 relaxation times. This difference is much larger in ^{1}H than in ^{13}C making it easier to leverage the differing ^{1}H relaxation times.

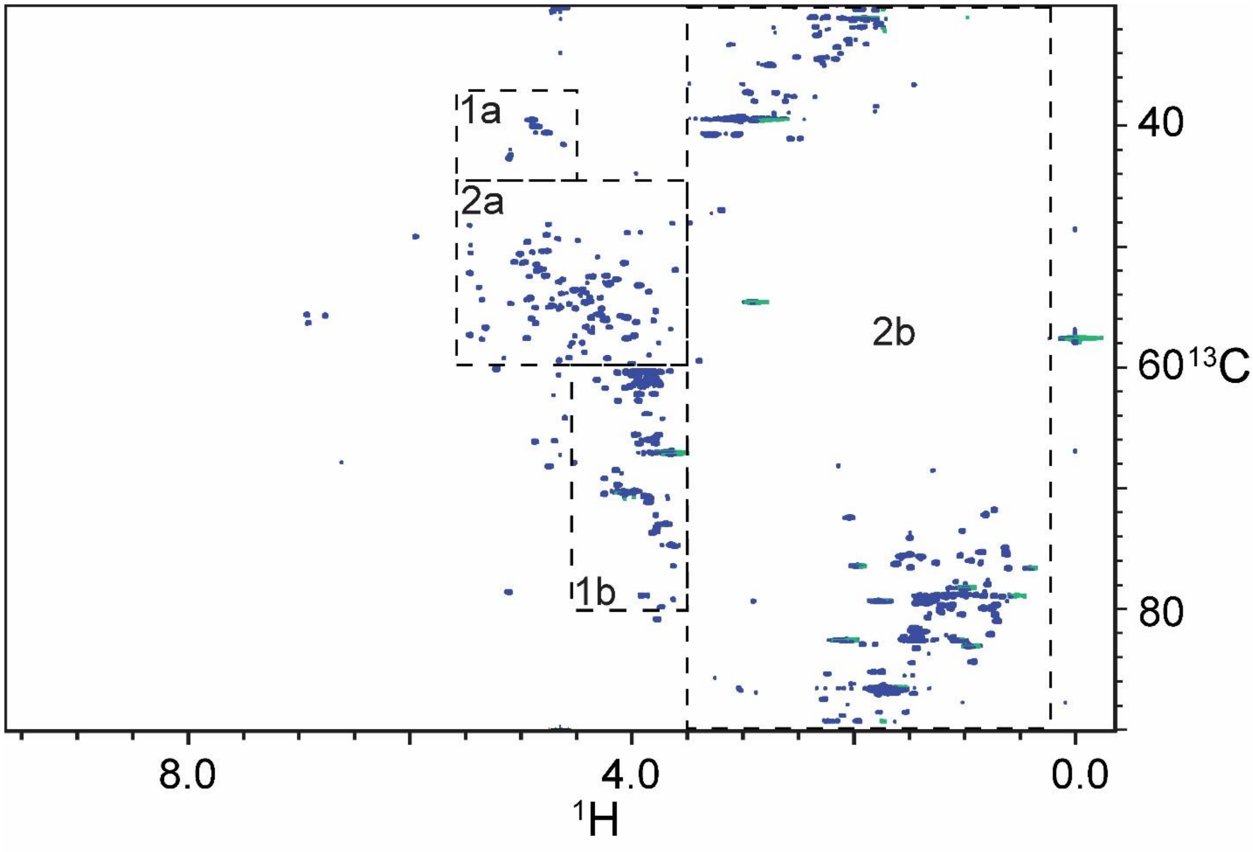

Figure S5: Binned intensity regions used in Table 1 for the ^{1}H-^{13}C HSQC and HSQC-TOCSY of RNase B Man5.

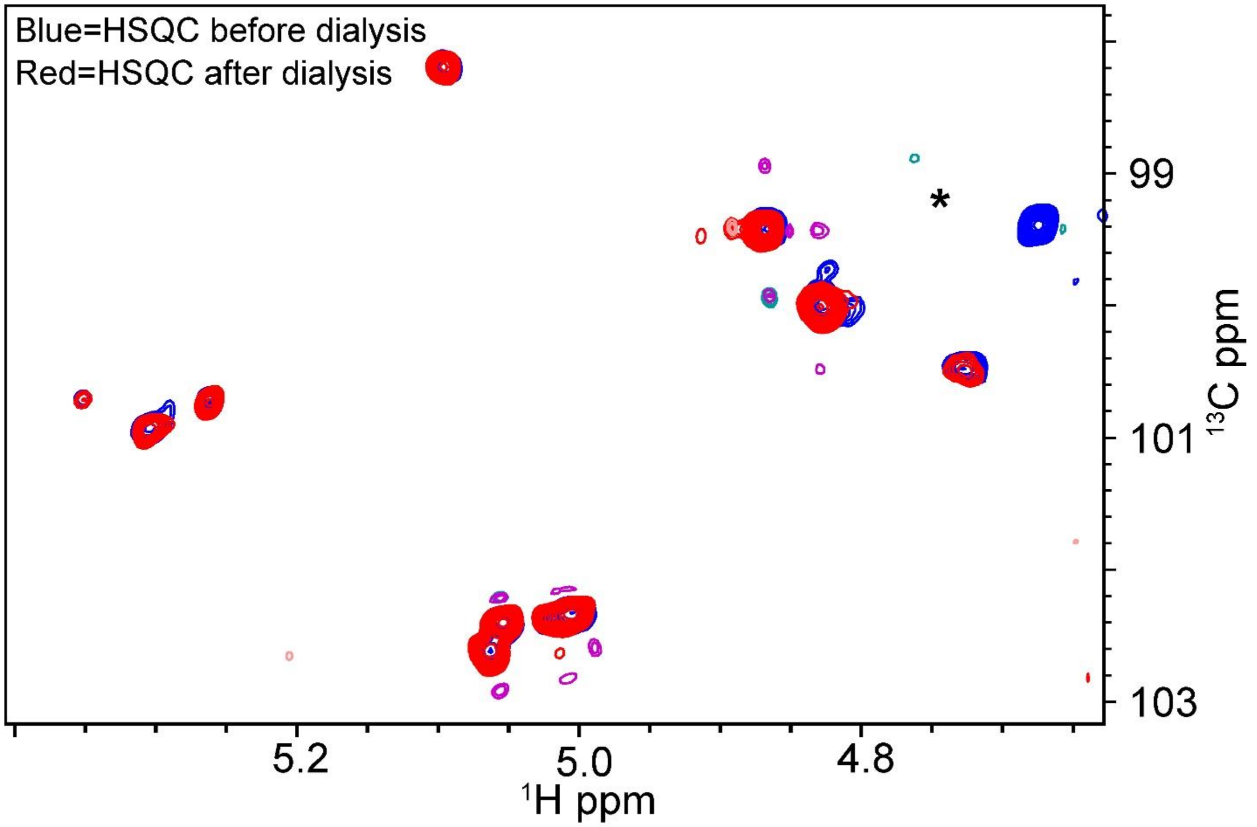

Figure S6: Overlay of ^{1}H-^{13}C HSQC anomeric region of vendor 1 RNase B before (blue) and after (red) dialysis. Asterisk highlights glycoside-like contaminant peak that was removed after dialysis.

Table S1. Linewidths and estimated T_2 relaxation values for regions of the spectrum belonging to protein alphas and glycan anomeric/ring ^{1}H-^{13}C correlations.

**Glycan Frequency[ppm]**		**Δν1/2 [Hz]**		**T_2 (ms)**	
**^{13}C**	**^{1}H**	**^{13}C**	**^{1}H**	**^{13}C**	**^{1}H**
79.2	3.62	26.23	11.90	12.14	26.76
76.4	3.62	25.88	16.10	12.31	19.78
69.2	4.25	24.47	22.40	13.01	14.21
79.8	3.73	24.82	21.70	12.83	14.67
74.7	3.62	18.66	16.10	17.07	19.78
68.4	4.15	25.17	22.40	12.65	14.21
72.2	3.78	23.59	14.00	13.50	22.74
80.8	3.77	22.36	16.80	14.24	18.95
74.6	3.64	23.77	19.60	13.40	16.25
73.5	3.77	22.36	7.00	14.24	45.49
73.7	3.81	21.48	17.50	14.83	18.19
73.5	3.78	21.30	12.60	14.95	25.27
71.1	3.85	19.37	10.50	16.45	30.32
78.8	3.91	21.30	14.70	14.95	21.66
70.8	3.84	21.30	7.00	14.95	45.49
72.9	3.69	21.48	14.00	14.83	22.74
70.8	3.83	22.89	10.50	13.92	30.32
70.4	4.24	21.30	9.80	14.95	32.49
70.6	3.88	21.48	16.10	14.83	19.78
70.2	4.08	18.49	7.70	17.23	41.35
69.7	4.13	21.30	9.80	14.95	32.49
70.2	3.98	21.30	9.10	14.95	34.99
70.4	4.06	22.18	8.40	14.36	37.91
39.5	4.91	21.13	7.70	15.08	41.35
42.4	5.10	20.42	7.00	15.59	45.49
42.6	5.11	24.65	7.70	12.92	41.35
40.5	4.77	21.83	9.10	14.59	34.99
40.0	4.87	21.65	8.40	14.71	37.91
			Average	14.44	28.82

**alphas Frequency[ppm]**		**Δν1/2 [Hz]**		**T_2 (ms)**	
**^{13}C**	**^{1}H**	**^{13}C**	**^{1}H**	**^{13}C**	**^{1}H**
52.7	4.61	29.22	22.40	10.90	14.21
49.0	4.75	31.34	29.41	10.16	10.83
52.4	4.76	30.46	23.80	10.46	13.38
51.4	5.00	26.94	24.50	11.82	13.00
50.3	4.83	32.22	18.20	9.89	17.50
50.3	4.75	33.80	21.70	9.42	14.67
51.2	5.07	28.52	25.90	11.17	12.29
50.3	4.81	24.65	12.60	12.92	25.27
49.3	4.68	25.17	16.10	12.65	19.78

52.4	4.93	31.51	20.30	10.11	15.69
52.9	4.66	29.22	17.50	10.90	18.19
52.9	4.67	23.94	17.50	13.30	18.19
50.4	4.90	26.58	9.10	11.98	34.99
52.7	4.64	29.05	30.81	10.96	10.34
50.3	4.77	28.34	25.90	11.24	12.29
50.3	4.77	30.10	21.70	10.58	14.67
51.2	5.06	24.47	18.90	13.01	16.85
48.1	4.75	25.00	20.30	12.74	15.69
51.5	4.86	30.28	27.31	10.52	11.66
51.8	4.83	29.22	28.01	10.90	11.37
51.8	4.83	25.53	16.10	12.48	19.78
49.0	4.78	24.29	18.20	13.11	17.50
51.2	4.97	26.06	18.90	12.22	16.85
52.0	4.86	27.46	18.90	11.60	16.85
50.6	5.02	24.65	19.60	12.92	16.25
49.6	4.94	25.53	18.20	12.48	17.50
			Average	11.55	16.37
